# A comprehensive review of animal models for cancer cachexia: Implications for translational research

**DOI:** 10.1016/j.gendis.2023.101080

**Published:** 2023-09-13

**Authors:** Li Li, Junaid Wazir, Zhiqiang Huang, Yong Wang, Hongwei Wang

**Affiliations:** a State Key Laboratory of Analytical Chemistry for Life Science, Medical School, Nanjing University, Nanjing, Jiangsu 210093, China; b Center for Translational Medicine and Jiangsu Key Laboratory of Molecular Medicine, Medical School, Nanjing University, Nanjing, Jiangsu 210093, China

**Keywords:** Animal model, Cancer cachexia, Muscle atrophy, Preclinical model, Weight loss

## Abstract

Cancer cachexia is a multifactorial syndrome characterized by progressive weight loss and a disease process that nutritional support cannot reverse. Although progress has been made in preclinical research, there is still a long way to go in translating research findings into clinical practice. One of the main reasons for this is that existing preclinical models do not fully replicate the conditions seen in clinical patients. Therefore, it is important to understand the characteristics of existing preclinical models of cancer cachexia and pay close attention to the latest developments in preclinical models. The main models of cancer cachexia used in current research are allogeneic and xenograft models, genetically engineered mouse models, chemotherapy drug-induced models, Chinese medicine spleen deficiency models, zebrafish and Drosophila models, and cellular models. This review aims to revisit and summarize the commonly used animal models of cancer cachexia by evaluating existing preclinical models, to provide tools and support for translational medicine research.

## Introduction

Cancer cachexia is a multifactorial metabolic syndrome characterized by progressive weight loss (skeletal muscle atrophy and fat degradation) and a negative nitrogen balance of nutrients (less anabolic than catabolic).^1^^,^^2^ The incidence of cancer cachexia varies according to the type of tumor, with a prevalence of up to 80% in some solid tumors, and is a direct cause of death in 20% of cancer patients.^3^ The development of cachexia not only seriously affects patients' quality of life but also greatly reduces their tolerance to radiotherapy and clinical outcomes, increases the chance of treatment-related complications, and can also induce infections and multiorgan dysfunction, leading to death.^4^ Despite numerous clinical trials in the field of oncologic cachexia, there is still no FDA-approved drug or treatment,^5^ and only anamorelin is currently approved in Japan for the treatment of oncologic cachexia.^6^ One reason for this dilemma is the apparent failure of translation from preclinical to clinical studies,^7^ making the selection of an appropriate model crucial. Various preclinical models of cancer cachexia differ in terms of the rate and size of tumor growth, the development of metastases, and the overall dynamics of the depletion process.^8^ Therefore, the selection of an appropriate animal model for the study of the pathogenesis of cancer cachexia is crucial for the prevention and treatment of cachexia, as well as for drug development.

## Animal models for inoculated grafts

### Allograft models

The allograft model is the most widely used model in preclinical studies of cancer cachexia. This model is created by subcutaneously, intramuscularly, or intraperitoneally injecting a certain number of rodent cancer cell lines into the same strain of mice, usually normal immunocompetent strains such as BALB/c and C57BL/6, to produce reproducible and fast-growing tumors that can lead to cancer cachexia.^9^ Such models include the Lewis lung cancer model (LLC),^10^^,^^11^ the C26 colorectal cancer model (C26),^12^^,^^13^ the Mac16 colon cancer model (Mac16),^14^ the Walker 256 rat breast cancer model (Walker 256),^15^ and the Yoshida liver cancer model (AH-130).^16^, ^17^, ^18^, ^19^

The LLC cachectic mouse animal model is modeled by subcutaneous inoculation of LLC cells into C57BL/6 mice unilaterally, with a typical inoculation rate of 5 × 10^5^–1 × 10^6^ cells per animal.^10^^,^^11^ This tumor induces rapid body and tissue depletion, with mice typically showing significant body weight loss 14 days after inoculation. At the molecular pathological level, the analysis revealed degraded and reduced mitochondria, significant upregulation of reactive oxygen species (ROS), a significant reduction in muscle cross-sectional area, and inhibition of myogenic signaling pathways in mice. Four weeks after inoculation, mice showed a decrease in protein synthesis and a significant increase in protein ubiquitination and autophagy.^20^ The disadvantage of the LLC cachexia model is that the tumors grow rapidly and are large in size, with an anorexia nervosa phenotype appearing only in the late stages.^21^ In addition, LLC cells frequently metastasize,^22^ and although the metastatic properties are similar to those of human cancer, it may be a confounding factor when anticancer drugs are used to treat cachexia.^9^

The animal model of C26 cachexia is modeled by subcutaneous inoculation of C26 cells into BALB/c mice unilaterally at a typical inoculation rate of 5 × 10^5^–1 × 10^6^ cells per mouse.^12^^,^^13^ The severity of C26-induced cachexia depends on the number of cell passages, the inoculation volume, and the injection site.^23^^,^^24^ Anorexia does not occur when C26 cells are injected subcutaneously or intramuscularly, whereas it occurs when they are injected intraperitoneally.^23^^,^^25^ The main disadvantage of this model is the rapid development of tumor malignancy, with only a few days between the appearance of malignancy and the death of the animal, which limits the therapeutic window for researchers. The advantage is that increased body consumption occurs at 2% of the total body weight of mice,^22^ which is similar to human cancer.^9^ Three weeks after inoculation of cells, mice lost weight, had reduced mitochondrial content in skeletal muscle, and decreased maximal mitochondrial respiratory capacity,^13^ a mitochondrial abnormality possibly caused by upregulation of pyruvate dehydrogenase kinase 4 (PDK4).^26^ Treatment of a C26 mouse model with the mitochondrial metabolic reprogramming agent trimetazidine improves oxidative metabolism and mitochondrial biogenesis through the upregulation of PGC1α.^8^ In animal models of C26 cachexia, systemic inflammation is an important factor contributing to cancer cachexia, the main drivers of which are generally IL-6 and leukemia inhibitory factor (LIF).^27^, ^28^, ^29^ In cancer patients with particularly high IL-6 levels, there is a clinical benefit of blocking IL-6 with TocilizuMab.^30^^,^^31^ The HSP90 inhibitors 17DMAG and PU-H71 also attenuate muscle atrophy in the C26 mouse model or myotubular atrophy in C2C12 cells induced by C26 conditioned medium.^32^ It has also been shown that the gene expression profile in the muscle of cachectic mice inoculated with C26 or LLC tumors differs from that in muscle biopsies from five patients with cachectic pancreatic cancer, which may be due to the heterogeneity of the tumors, each of which may have a different secretome and consequently a different gene expression profile; therefore, more research is needed.^33^

The Walker 256 cachexia rat model is modeled by subcutaneous inoculation of Walker 256 tumor cells into rats unilaterally at a rate of 2 × 10^6^–2 × 10^7^ cells per animal.^15^ The model has several key features of cachexia, including decreased body weight, reduced skeletal muscle and adipose tissue, decreased food intake, and the presence of a systemic inflammatory response.^34^ Liver metabolism is impaired in Walker 256 tumor cachexia and is more severe in the cachexia model in weaned rats^15^; administration of high doses of insulin increases p-Akt levels in the adipose tissue of rats with tumors, decreases p-HSL levels, and inhibits adiposity and weight loss in rats with tumors, demonstrating that activation of Akt is a potential strategy to prevent adiposity in cancer cachexia.^34^ Its main drawback is the excessive tumor burden,^35^ with tumor weights of up to 19 g^36^ in cachexia and tumor loads of up to 50% of body weight.^21^ However, tumor mass in humans is usually less than 1% of body weight, making the Walker 256 model ethically problematic.^8^

The Yoshida AH-130 cachexia rat model was modeled by intraperitoneal injection of Yoshida ascites hepatocellular carcinoma AH-130 cells into male Wistar rats at an inoculation rate of 1 × 10^8^ cells per rat.^16^, ^17^, ^18^, ^19^ This rat model of liver cancer is rapidly modeled and reproducible,^8^ and tumor growth leads to severe anorexia and wasting and a 30% weight loss 14–16 days after inoculation, eventually leading to death.^37^ Similar to the C26 model, cachexia occurs when the tumor load is small.^21^ AH-130 rats exhibit reduced systemic IGF-1, and preexercise fails to suppress systemic IGF-1 production.^38^ The use of a small molecule agent, MT-102, reduces catabolism and activates the PI3K/Akt/mTOR pathway to induce skeletal muscle anabolism, thereby improving survival in AH-130 cachectic mice.^16^ However, the significant disadvantage of this model is that it is highly aggressive, and in the late stages of cachexia, large amounts of ascites can develop, thereby compressing the organs.

A recently developed mouse fibrosarcoma model has recently been developed to study cachexia in C57BL/6 mice. The cachectic CHX207 fibrosarcoma cells evolved from the noncachectic MCA207 fibrosarcoma cells, and the two cells can be compared between cachectic and noncachectic mice with the same tumor type. Thirteen days after inoculation with tumor cells, CHX207 mice exhibited key clinical features of cancer cachexia, including systemic inflammation, increased plasma IL-6 concentrations, increased energy expenditure, adipose tissue loss, skeletal muscle wasting, and weight loss.^39^

### Xenograft models

Xenografts use human-derived tissues or cells in a state that more closely resembles human tumors but require immunodeficient mice as hosts. The cell-line-derived xenograft (CDX) transplant mouse model is constructed by inoculating a human-derived cell line into the animal.^40^ In addition, the patient-derived xenograft (PDX) transplant mouse model is constructed by transplanting patient-derived tumor tissue into the host animal.^41^, ^42^, ^43^

One of the CDX transplantation animal models is the intraperitoneal injection of 1 × 10^7^ ES-2 human ovarian cancer cells into Nod SCID-gamma (NSG) (NOD-scid/IL2Rg^null^) immunodeficient mice, which after approximately 2 weeks develop abdominal tumors that infiltrate the omentum, mesentery, and adjacent organs. It results in decreased bone mineral density and bone mineral content, increased levels of IL-6 in plasma and ascites, increased levels of p-STAT3 in skeletal muscle, decreased levels of p-Akt, decreased mitochondrial proteins, and elevated levels of ubiquitination. Conditioned medium with ES-2 cells directly induced myotubular atrophy in C2C12 cells, accompanied by increased activation of the JAK2/STAT3 signaling pathway. This provides a suitable model for the study and targeted treatment of ovarian cancer-associated cachexia.^44^

The HT1080 human fibrosarcoma cell line secretes several cachexia-related factors, such as growth differentiation factor 15 (GDF 15), interleukin 6 (IL-6), and activin A, and it has been reported that this tumor model has a similar phenotype to cancer cachexia.^45^ The model was developed by subcutaneously injecting 5 × 10^6^ HT-1080 tumor cells into female SCID ICR-Prkdc mice. This model reflects the energy imbalance, impaired muscle function and atrophy, increased inflammatory factors, and reduced mobility that characterize human cachexia.

Its limitations are that fibrosarcoma is a rare tumor type, and although accompanied by elevated levels of multiple cytokines, immunodeficient mice do not truly reflect the immune characteristics of human cachexia; tumor development is rapid and does not recapitulate the tumor microenvironment of human cancer; and HT1080 cells are insensitive to chemotherapeutic agents and therefore cannot be studied in combination with chemotherapy or in long-term cachexia intervention experiments.^46^

PDX transplantation mouse models are constructed in which a first generation of transplanted tumors can be formed after patient-derived tumor tissue has been implanted and adapted to the new host. After it has grown to a certain size, the tumor is removed and inoculated into a new batch of mice for culture, producing a population of animals with the same tumor for preclinical studies of treatment efficacy.^47^ In recent years, attempts have been made to use the PDX model in the field of cancer cachexia research. Fresh pancreatic cancer tissues from two patients with pancreatic cancer, both in a cachectic state, were inoculated into two immunodeficient mice and then removed and inoculated into the unilateral flanks of five mice to construct the PDX model after the formation of first-generation transplants. The results showed that the PDX model mice showed significant decreases in the weight of the tumor, common skeletal muscle groups such as the anterior tibial and quadriceps muscles, and the heart and that the degree of weight loss was not directly related to tumor size, which was highly compatible with the clinical presentation of patients with clinical cachexia.^48^ Pathological analysis revealed that the expression levels of muscle atrophy-related genes (Foxo1, Socs3, Stat3, Acvr2b, Atrogin-1, and Murf1) were also significantly upregulated in the PDX group of mice. In addition, the expression profiles of cytokines in the spleen and tumor microenvironment of the PDX mice were significantly altered compared to those of the control group, and the expression profiles of cytokines in the tumor microenvironment were significantly different between the two PDX groups, further confirming the existence of tumor heterogeneity among pancreatic cancer patients. These data indicate that the PDX model can be used as an important tool to study the pathogenesis of cachexia and to screen for molecular markers such as soluble metabolic regulatory proteins. The use of the PDX model can further elucidate which cytokines are associated with the pathogenesis of cachexia, thus providing a potential therapeutic target for immunotherapy (Table 1).^48^

**Table 1:** The inoculated graft model of cancer cachexia.

Inoculated graft model	Strain	Cell	Cell numbers per animal	Mouse age	Inoculation method	References
Allograft models	C57BL/6	LLC	5 × 10^5^–1 × 10^6^ cells	6–8 weeks	Subcutaneously	10,11
	BALB/c CD2F1	C26	5 × 10^5^–1 × 10^6^ cells	6–8 weeks	Subcutaneously	12,13
	Wistar rats	Walker 256	2 × 10^6^–2 × 10^7^ cells	90 days	Subcutaneously	15
	Wistar rats	AH-130	10^8^ cells	7 weeks	Intraperitoneally	16, 17, 18, 19
	C57BL/6	CHX307	1 × 10^6^ cells	10–11-weeks	Intramuscularly	39
Xenograft models	NSG mice	ES-2	1 × 10^7^ cells		Intraperitoneally	44
	ICR-Prkdc mice	HT-1080	5 × 10^6^ cells or 5 × 10^6^ cells	8–12 weeks	Subcutaneously or heterotopically	45,46

NSG mice: Nod SCID gamma mice.

## Genetically engineered animal models

### Apc^Min/+^ mouse model

Apc^Min/+^ mice were first screened in 1990 in the Dove laboratory at Wisconsin-Madison University.^49^ The adenomatous polyposis coli (Apc) gene is an important oncogene in the Wnt signaling pathway and plays an important role in the development of colorectal cancer. This mouse is known as multiple intestinal neoplasia (Min) due to a nonsense mutation in codon 850 of one of the strands of the Apc gene, where the codon encoding leucine (TTG) is converted to a stop codon (TAG), resulting in premature termination of protein translation and preventing the oncogene Apc from functioning, thus causing multiple adenomas in the intestine. Purebred embryos of Apc^(Min)^ mice are lethal, and low-aged heterozygous mice can grow more than 30 adenomas throughout the intestine, with most dying 120 days after birth. This model exhibits a slow progression of development similar to that of human cachexia. Mice develop colon cancer at 4 weeks of age, begin to lose weight at 14–20 weeks of age, and lose more than 15% of their body weight at 20 weeks of age.^49^^,^^50^ The cachectic phenotype of this model is associated with IL-6-induced Atrogin1 expression, inhibition of mTOR, and intestinal barrier dysfunction.^50^, ^51^, ^52^ In Apc^Min/+^ mice, mechanosensitive signaling is maintained in mouse skeletal muscle, but the chronic inflammatory signaling pathway STAT3/NF-κB signaling attenuates eccentric contraction-induced protein synthesis^53^; CGRPPBN neurons are activated in this model of mice, and inactivation of CGRPPBN neurons increases feeding and thus counteracts weight loss, revealing that CGRPPBN neurons play an important role in the development of the Apc^Min/+^ cachexia animal model.^54^ Using a 23-week-old Apc^Min/+^ model, it was found that hypothalamic‒pituitary‒adrenal axis activity is enhanced during cachexia and is associated with increased glucocorticoid concentrations in serum, skeletal muscle and liver and its regulatory role in the transcriptional regulation of skeletal muscle catabolism and hepatic metabolism.^55^ It has also been claimed that Baoyuan Jiedu decoction alleviates cancer cachexia-induced muscle atrophy by modulating mitochondrial function in Apc^Min/+^ mice.^56^

### KPC mouse model

The KPC (KrasG12D/+; Trp53R172H/+; Pdx-1-Cre) mouse model is a pancreatic ductal adenocarcinoma model in which KrasG12D is an activating mutant of the oncogene Kras and Trp53R172H is a suppressor mutant of p53.^57^ Both genes contain Loxp–Stop–Loxp termination sequences upstream, and double transgenic mice were prepared by transferring the two gene constructs into the mouse genome. The genes are not transcribed due to the termination sequence. Pancreatic duodenal homology frame 1 (PDX-1) is a pancreas-specific transcription factor that is expressed early in embryonic development.^58^ After double genetically modified mice are mated with Pdx1-Cre mice, Cre recombinase is expressed in the pancreas in a tissue-specific manner, and the mutant begins to be expressed after excision of the termination sequence, leading to pancreatic cancer in mice with metastasis within 2–3 months.^59^ This results in the Kras gene being active before birth in KPC mice, a process that takes at least 10 years for human pancreatic cancer to develop, but does not suggest that in humans, the Kras mutation is active before birth.^60^ A new model of KPC cell homotransplantation was developed whereby KPC cell lines were isolated from pancreatic cancer lesions in KPC mice and inoculated subcutaneously, peritoneally, or in situ into mice for modeling. The results showed that all three injection methods could cause tumor malignancy, but the in situ and peritoneal routes were more likely to induce a severe malignant phenotype than subcutaneous inoculation.^59^

### The KPP mouse model

In the KPC mouse animal model, Kras is active before birth, which differs from humans,^60^ and this model was improved by retaining the Kras G12D mutant in KPC mice, replacing the p53 gene with the oncogene Pten, which contains Loxp sequences on both sides of the Pten allele, and replacing the promoter PDX1, which drives Cre recombinase, with Ptfla, which is still active in the adult pancreas. Because the mice exhibit the genotypes Kras +/G12D, Pten f/f, and Ptf1a +/ER-Cre, they are named KPP mice.^33^ The advantage of KPP mice as a tumor cachexia model is that it allows for the induction of pancreatic cancer and its associated cachexia after the mice have finished growing, which better mimics muscle and adipose tissue depletion. The KPP also has a relatively longer time for tumor development than, for example, the commonly used C26 model and therefore has more opportunities for intervention studies. The characteristics of KPP mice also better reflect the clinical signs common to human cachexia compared to traditional models.^33^

### ASV-B transgenic hepatocellular carcinoma cachexia

Systemic inflammation is considered a key driver of cancer cachexia, yet anti-inflammatory drugs have not been effective in clinical studies of cachexia. To address this paradox, researchers crossed transgenic HCC mouse models with mice harboring defects in myeloid-mediated inflammation to investigate the functional importance of innate immune cells for hepatocellular carcinoma (HCC)-associated cachexia.^61^ The transgenic ASV-B mouse strain is a well-established HCC model that achieves specific expression of the SV40 large T oncogene protein in male mouse hepatocytes via the anticoagulant III promoter.^62^ Hypoplastic hepatocytes appeared at 8 weeks, hepatic adenomas at 12 weeks, and HCC at 16 weeks of age.^63^ The resulting ASV-B mice exhibited various cachectic features, such as weight loss, reduced skeletal and cardiac muscle and adipose tissue, increased expression of proinflammatory cytokines in the blood, and anemia. It has been shown that 50% of mice carrying diethylnitrosamine-induced HCC show signs of cachexia at 16–18 months of age,^64^ but the long duration and 50% epizootic rate limit the widespread use of this model. In contrast, cachexia was detected in 12-week-old ASV-B mice at 100% ectopic rate. Thus, the ASV-B model is a powerful complement to existing models and can provide a better understanding of the mechanistic basis of hepatocellular carcinoma-associated cachexia.^61^

### The Pik3ca∗ mouse model

Pik3ca is a protooncogene whose abnormal expression is associated with the development, infiltration, and metastasis of a variety of tumors, including breast, colorectal, lung, ovarian, and esophageal cancers.^65^ Transgenic female mouse models of ovarian tumors with oocyte-specific expression of constantly active PI3K (Pik3ca∗) were generated by crossing pure female mice with Cre-inducible knock-in Pik3ca∗ alleles with heterozygous *Gdf9-iCre*^*+/−*^ males to investigate the initiation and progression of tumor malignancy.^66^^,^^67^ Using this model, we found that the symptoms of cachexia in this mouse were similar to those of human cachexia progression, including dramatic weight loss, skeletal muscle atrophy, and adipose tissue depletion. Two cachexia biomarkers, activin A and GDF15, were significantly increased in mouse serum; protein hydrolysis was hyperactivated in muscle, muscle-specific E3 ligases Atrogin-1 and Murf-1 were significantly upregulated, and the cross-sectional area of muscle fibers was reduced; adipose tissue was significantly lost, uncoupling protein 1 (Ucp1) was upregulated and was accompanied by adenofibrosis. This mouse model may be one of the most suitable preclinical models.^66^

### Other knockout mouse models

In addition, we are also concerned about the study of a certain gene knockout mouse in a cachexia disease model, and we summarize some of them here. Acetyl coenzyme A synthase short chain family member 2 (Acss2) is an acetyl coenzyme A synthase that promotes lipid synthesis and epigenetic reprogramming, while in a tumor context, metabolic stress in the body induces Acss2 expression, which is associated with poor prognosis in pancreatic cancer. The use of Acss2 knockout mice revealed that knockdown of Acss2 attenuated muscle wasting and prolonged the survival time of in situ transplanted mice.^68^ Lipocalin 2 (Lcn2) was recently identified as an endogenous ligand for the melanocortin type 4 receptor (Mc4r), a key regulator of appetite. In a mouse model of pancreatic cancer, Lcn2 is significantly upregulated, its expression correlates with reduced food intake, and Lcn2 deficiency contributes to the prevention of cachexia anorexia.^69^ In addition, lipocalin 2 knockout mice (Lcn2-KO) had reduced ATGL expression in iWAT compared to tumor-loaded WT mice, as well as reduced expression of the myasthenic molecular markers MuRF-1 and Atrogin1.^70^ Cathepsin K (Ctsk) is a widely expressed cysteine protease with upregulated expression in the cathepsin family during multiple forms of skeletal muscle atrophy. Using Ctsk knockout mice, it was found that knockdown of Ctsk alleviated IRS1 degradation, skeletal muscle mass loss, and muscle dysfunction in a mouse model of LLC cachexia.^71^ DNA damage and developmental regulation 1 (Redd1) is a stress response protein that inhibits mTORC1 (rapamycin targeting mechanism 1 complex). Treatment of C2C12 myotubes with LLC medium increases Redd1 mRNA expression and reduces myotube diameter. To investigate the role of Redd1 in cancer cachexia, we used 12-week-old male wild-type or systemic Redd1 knockout (Redd1 KO) mice inoculated with LLC cells and euthanized 28 days later. In LLC-induced cancer cachexia, skeletal muscle Redd1 expression was increased, Akt and 4E-BP1 phosphorylation was unaffected in muscle from knockout Redd1 mice, mTORC1 activity was maintained, and dephosphorylation of muscle Foxo3a was inhibited, suggesting that knockout Redd1 prevents loss of body weight and lean tissue mass by regulating protein synthesis and degradation pathways, but not fat mass, but did not affect fat mass.^72^ Inducible nitric oxide synthase (iNOS; NOS2) is highly expressed in cachectic muscle and is a known downstream effector of the NF-κB pathway. iNOS knockout mice and mice treated with the clinically tested iNOS inhibitor GW274150 were protected from muscle depletion in both sepsis and tumor cachexia models. It was further demonstrated that iNOS causes muscle atrophy by disrupting mitochondrial content, morphology, and energy-generating processes such as the TCA cycle and acylcarnitine transport. However, all these effects can be reversed by the iNOS inhibitor GW274150, and these results offer the possibility of a treatment for cachexia (Table 2).^73^

**Table 2:** The genetically engineered models of cancer cachexia.

Genetically engineered models	Strain	Gene	Mouse age	References
Apc^Min/+^ mouse model	C57BL/6	Apc^Min/+^	14–23 weeks	49, 50, 51, 52, 53, 54, 55, 56
KPC mouse model	C57BL/6	Kras^G12D/+^; Trp53^R172H/+^; Pdx^Cre/+^	7–12 weeks	59
KPP mouse model	C57BL/6	Kras^G12D/+^; Pten^f/f^; Ptf1a^+/ER-Cre^	–	33,60
ASV-B mouse model	C57BL/6	SV40 large T; Hif1a^f/f^	12 weeks	61, 62, 63, 64
Pik3ca∗ mouse model	C57BL/6	PIK3CA; Gdf9-^iCre+/−^	–	65, 66, 67
Other gene knockout models	–	Acss2	–	68
	C57BL/6	Lcn2	7–10 weeks	69,70
	C57BL/6	Ctsk	9 weeks	71
	C57BL/6	Redd1	12 weeks	72
	C57BL/6	Nos2	8–12 weeks	73

## Chemically induced animal models

In addition to factors associated with cancer, chemotherapy can also lead to wasting and the subsequent development of cachexia.^74^ Chemotherapy has anticancer effects, but due to its nonspecific cytotoxicity, it can also have harmful off-target side effects on healthy cells. It may affect muscle mass through enhanced protein hydrolysis, leading to muscle weakness and thus worsening the overall quality of life of patients.^75^^,^^76^ Current research on the effects of chemotherapeutic agents on skeletal muscle has focused on three chemotherapeutic agents: anthracycline doxorubicin (DOX), the platinum-based alkylating agent cisplatin (CDDP), and the antimetabolite 5-fluorouracil (5-FU).^77^

### Animal model of DOX-induced atrophy in cachectic skeletal muscle

Anthracyclines are commonly used therapeutic agents for many types of cancer,^78^ with one of the most frequently used anthracyclines being doxorubicin, also known as adriamycin (DOX), due to its strong cytotoxic effects.^79^ However, while DOX is effective in treating cancer, it can also damage healthy tissue, particularly cardiac and skeletal muscle, increasing the risk of morbidity and mortality.^80^ Therefore, in studies of cachexia, researchers will use adriamycin to construct a mouse model of cachexia by administering adriamycin (4 mg/kg in 0.9% NaCl) intraperitoneally to mice on three occasions (i.e., days 1, 3, and 5) over a 7-day period to induce the adriamycin cachexia mouse model.^78^

### Animal model of cisplatin-induced atrophy of cachectic skeletal muscle

Cisplatin (CDDP) is a platinum-based alkylating agent that enhances DNA damage by forming highly reactive monohydrate complexes.^79^ It may induce inflammation by increasing peroxidase (PRX) sulfonylation to promote reactive oxygen species (ROS) production and NF-κB signaling activation, leading to cachectic muscle degradation.^74^ Intraperitoneal administration of 6 mg/kg cisplatin to adult male Lister-hooded rats induces a pathological phenotype that produces acute cachexia.^80^ Cisplatin-induced cachexia dysregulates hypothalamic and systemic adiposity, and this effect is antagonized by cannabidiol, attenuating cachexia-induced weight loss.^80^

### Animal model of 5-fluorouracil (5-FU)-induced atrophy of cachectic skeletal muscle

5-FU is an antimetabolite class of chemotherapeutic agents mainly used in the treatment of colorectal cancer that causes cytotoxicity by misincorporating nucleotides into RNA and DNA or inhibiting nucleotide enzyme thymidylate synthase activity, which in turn leads to nucleic acid damage as well as cell cycle arrest.^81^ It has been shown that the 5-FU-based combination regimen FOLFIRI (5-FU, folinic acid, and irinotecan) can cause cachexia by activating the MAPK pathway, increasing ROS levels, and reducing the number and size of mitochondria^74^^,^^82^, ^83^, ^84^, ^85^ and is modeled by twice weekly intraperitoneal injections of FOLFIRI (5-FU: 50 mg/kg; folinic acid: 90 mg/kg; irinotecan: 24 mg/kg) to induce a cachectic phenotype.^86^

In conclusion, the effect of chemotherapy on muscle wasting should be taken into account when building preclinical models of cancer cachexia to more realistically reflect the onset and progression of cancer cachexia, and models lacking chemotherapy use may underestimate the role chemotherapy plays in cancer cachexia and lead to conversion failure (Table 3).

**Table 3:** Chemically induced animal models of cancer cachexia.

Chemically induced animal models	Strain	Chemical drugs	Dose	Inoculation method	References
DOX-induced atrophy	BALB/c	Doxorubicin	4 mg/kg	Intraperitoneally	78
Cisplatin-induced atrophy	Lister hooded rats	Cisplatin	6 mg/kg	Intraperitoneally	80
5-FU-induced atrophy	CD2F1	5-Fluorouracil	50 mg/kg	Intraperitoneally	86
		Leucovorin	90 mg/kg		
		CPT-11	24 mg/kg		

## Other animal models

### Spleen deficiency cancer cachexia model

Based on the basic theory of Chinese medicine, domestic scholars have established a combined animal model of spleen deficiency cancer cachexia. The modeling method was to use the three-factor compound method (bitter-cold diarrhea, fatigue injury to the spleen, and diet disorder) to induce a mouse model of deficiency disease (daily gavage of senna leaf decoction in the morning, swimming exhaustion experiment in the afternoon, half a day of feeding, half a day of free feeding and drinking). After the successful induction of the deficiency model, C26 cells were inoculated subcutaneously to construct a cancer cachexia model producing spleen deficiency.^87^

### Zebrafish and Drosophila cachexia models

In addition to the use of rodents, there have been studies using zebrafish and Drosophila to construct cachexia models. There are several common models of zebrafish cachexia, including a single oncogene-driven liver cancer model fabp10a: TetOn; tre:eGFP-kra^sv12^ [Tg(Ras)],^88^ a double transgenic model Tg(Myc&Ras),^89^ and a transgenic model expressing the oncogene xmrk Tg(fabp10.rtTA; TRE:xmrk; krt4:GFP), also known as TO(xmrk).^90^ All of these models can produce manifestations of cachexia, such as depletion of adipose and muscle tissue. Naser et al used isotope tracing techniques in adult zebrafish to study tumor-induced systemic metabolic changes at the molecular level and showed the presence of metabolic alterations in nontumor tissues to support tumor growth. They demonstrated that alanine produced from glucose is excreted from melanoma and translocated to the liver for gluconeogenesis. Pharmacological interference with this metabolite exchange could reduce tumor burden. Thus, zebrafish could serve as a new model to study the relationship between tumor and host metabolism and deepen our understanding of cancer-related diseases such as cachexia.^91^^,^^92^ In another study in a Ras- and Myc-driven zebrafish HCC model, disruption of leptin signaling and normal expression of Igf1 significantly rescued anorexia, muscle wasting, and adiposity.^89^ Another study showed that in zebrafish cachexia, the intestinal structure was progressively disrupted, manifested by inflammatory intestinal phenotypes such as villus damage, thinning of the intestinal wall, increased number of Golgi cells, reduced size of Golgi cells, and eosinophil infiltration. Analysis of intestinal gene expression by RNA-seq revealed dysregulation of genes related to intestinal function, epithelial barrier and homeostasis, and activation of pathways such as inflammation, epithelial mesenchymal transition, extracellular matrix organization, and hemostatic pathways. Gene set enrichment analysis revealed that the intestinal gene signature of liver tumor fish shares commonalities with human inflammatory bowel disease, and this study provides the first systematic characterization of intestinal disruption under liver tumor conditions and suggests targeting intestinal inflammation as a potential approach to manage cancer cachexia.^93^

Drosophila is another important model organism for the study of human diseases, as more than 75% of the genes associated with human diseases are retained in Drosophila.^94^ The following are several Drosophila models that produce a cachectic phenotype: One is the Yki^act^ model, which induces abnormal activation of the transcriptional coactivator yorkie (Yki) in intestinal stem cells. Yki is a homolog of the human oncogene YAP1, which leads to excessive proliferation of intestinal tumor cells and causes systemic organ depletion, including muscle dysfunction, fat loss, hyperglycemia, and even death.^95^, ^96^, ^97^ Another model is *Ras*^*V12*^ *scrib^−/−^*, which can induce malignancy in Drosophila by knocking down the tumor suppressor scribble (scrib) in the background and expressing oncogenic Ras (referred to as RasV12). Transplantation of *RasV12 scrib^−/−^* in tumor-eyed adult discs into adult Drosophila triggers organ depletion.^96^^,^^98^ Additionally, transplantation of tumor cells into larvae can cause physical depletion.^99^ Another model is *RasV12 Csk^−/−^*, achieved by expressing active Ras in the adult eye disc and knocking down C-terminal Src kinase (Csk). Since *RasV12 Csk^−/−^* tumors prefer to grow in larvae on a high-sugar diet, this tumor model relies on stimulation by a high-sugar diet.^100^ Another model is RasV12dlgRi, which uses the Ras gene while activating oncogenic RasV12 and knocking down the polarity protein disc-large (Dlg1) to induce tumors in Drosophila eye-tentacle epithelial discs.^101^ Another model is Pvrλ, where over-activation of Pvr by expressing the active form of the PDGF/VEGF receptor Pvr can lead to overgrowth of oculo-tactile epithelial tissue, and distal to the tumor, we observed muscle fiber detachment and decreased muscle/keratin ratio.^101^^,^^102^

Using the Ras^V12^dlg^Ri^ model, tumor-secreted matrix metalloproteinase 1 (Mmp1) was found to alter the availability of Gbb, a ligand for TGFβ, in tumors and trigger TGFβ activation in adiposomes, leading to disruption of basement membrane (BM)/extracellular matrix (ECM) proteins at intercellular adhesion sites in adipocytes. In addition, Mmp1 and Mmp2 act directly on adiposomes and muscles to trigger their destruction. In the absence of tumors, the disintegration of intercellular adhesions of fat bodies is sufficient to induce muscle detachment, suggesting that the disintegration of fat cells releases signals that eventually lead to muscle detachment. In conclusion, we gained valuable insights into the interactions between tumor, muscle and adipose tissue during wasting disorders.^101^ Using a Drosophila larval cachexia model, it was found that circulating levels of cholesterol ecdysteroids were decreased in cachexia, while overexpression of the cholesterol ecdysteroid transporter protein families Oatp74D and Oatp33Eb induced cholesterol ecdysteroid levels in tumors that exacerbated tumor cachexia but did not worsen it; in contrast, knocking down its expression in tumors reduced cachexia. In contrast, feeding food containing cholesterol ecdysteroids to cachectic animals improved cachexia. This phenomenon may be a way for tumors to induce cachexia symptoms by absorbing circulating steroids at the expense of healthy tissues.^103^ A study using the Yki^3SA^ Drosophila tumor model further revealed that the inhibition of antimicrobial enzyme PGRP-SC2 activity in the gut of tumor-bearing Drosophila and an increase in the number of commensal bacteria led to abnormal activation of NF-κB in the kidney, causing uric acid, and circulatory disturbances. In contrast, the tumor-derived cachexia factor Impl2 can exacerbate uric acid accumulation and host wasting and death independently of NF-κB activation in the Drosophila kidney.^104^ Another study used a Drosophila model to identify muscle secretory factors using transgenic screening techniques and validated them in mouse myotubular cells and found that mouse Fibcd1 is a conserved muscle factor that protects myofibers and functions through the Erk signaling pathway. Further experiments revealed that local injection of recombinant Fibcd1 could ameliorate muscle atrophy caused by tumors and block the transcriptional levels of muscle atrophy-related genes.^105^ Using the Yki^act^ Drosophila cachexia model, we found that the level of the tumor-secreted factor Impl2 was elevated in the Drosophila cachexia model, and Impl2 caused lipid imbalance, while knockdown of Impl2 restored both fat and muscle to normal levels.^106^ This provides a new technical avenue to explore complex multiorgan integrative diseases and demonstrates the value of low-level model animals for features and applications in tumor research (Table 4).

**Table 4:** Other animal models of cancer cachexia.

Other animal models	Genes or Treat	Other details	References
Spleen deficiency cachexia model	Limit feeding	The mice were offered food from 8 am to 8 pm daily but were allowed to drink freely	87
	Induce fatigue	Mice were forced to swim once a day until they were exhausted	
	Induce purging	Mice were treated with an aqueous extract of senna (20 μl/mg/day on days 1–16 and 28 μl/mg/day on days 17–30)	
Zebrafish	Tg(Ras)	Fabp10a:TetOn; tre:eGFP-krasv12	88
	Tg(Myc&Ras)	Cross Tg(Ras) with fabp10a:TetOn; tre:Myc [Tg(Myc)]	89
	TO(Xmrk)	fabp10:rtTA; TRE:xmrk; krt4:GFP	90
Drosophila	Yki^act^	Overexpression of the Yki gene in adult intestinal stem cells produces intestinal tumors and systemic organ atrophy.	95, 96, 97
	*Ras*^*V12*^*Scrib*^*−/−*^	The oncogenic gene RasV12 is expressed and the tumor suppressor Scrib mutated, thereby inducing cancer in Drosophila.	96,98,99
	*RasV12 Csk*^*−/−*^	The oncogenic gene RasV12 is expressed and the C-terminal Src kinase (CSK) is knocked down.	100
	*Ras*^*V12*^*dlg*^*Ri*^	The oncogenic gene RasV12 is expressed and the polarity protein disc-large (dlg1) is knocked down.	101
	Pvr^λ^	Expression of a constitutively active form, Pvrλ, resulted in the overactivation of the PDGF/VEGF receptor Pvr, leading to robust overgrowth of the eye-antennal epithelium.	101,102

### In vitro cellular models of cancer cachexia

Cell lines help to study diseases at the molecular level and are also good tools for the selection of therapeutic agents.^21^ In cancer cachexia, a common in vitro model is to treat differentiated C2C12 and L6 cells or primary myoblasts using the culture medium supernatant of cachectic tumor cells to induce tumor cachexia, or to model muscle cells after costimulation with cachectic factors TNFα and IFNγ or IL-6, TNFα and LIF.^107^, ^108^, ^109^, ^110^, ^111^, ^112^, ^113^ Traditional monolayer cell culture is widely used; however, this method does not reliably capture phenomena at the tissue level, such as myotube maturation, force production, extracellular matrix remodeling, and capillarization.^33^ Moreover, the signaling pathways and molecular pathways involved in the degradation of cachectic skeletal muscle in human skeletal muscle tissue may differ from those in experimental animals^33^^,^^114^; therefore, the development of in vitro models of human skeletal muscle degradation is important for studying the pathogenesis of cachexia, screening, identifying and tracking the best biomarkers and assessing therapeutic strategies.^114^ In recent years, in vitro culture models of 3D skeletal muscle have emerged and matured,^107^^,^^112^^,^^115^ and they can be used for drug screening by detecting changes in muscle contractility after exposure to drugs.^116^, ^117^, ^118^ A recent study using two 3D organoid culture systems (methylcellulose-based suspension droplet 3D cancer spheroids and classical matrix gel-based 3D organoid culture) to investigate the effect of pancreatic cancer cells on muscle cell metabolism showed that both conditioned media had the same phenotypic effects of cachexia as those observed in 2D cell culture, such as p38MAPK pathway activation, a significant upregulation of the Atrogin1 gene, and decreased myosin heavy chain (MyHC) expression.^119^ 3D organoids have the advantage that they can better mimic the physiological conditions in vivo compared to traditional 2D cell culture and will be relatively less time-consuming than mice, making them promising as preclinical models.

In addition to using muscle cells to study muscle atrophy, researchers have also used adipocytes treated with pro-atrophy factors to construct adipose degradation models to study adipose tissue atrophy in tumorous malignant masses and to elucidate the mechanisms of white fat and its browning in malignant masses.^120^, ^121^, ^122^

## Conclusion

**Figure 1:**
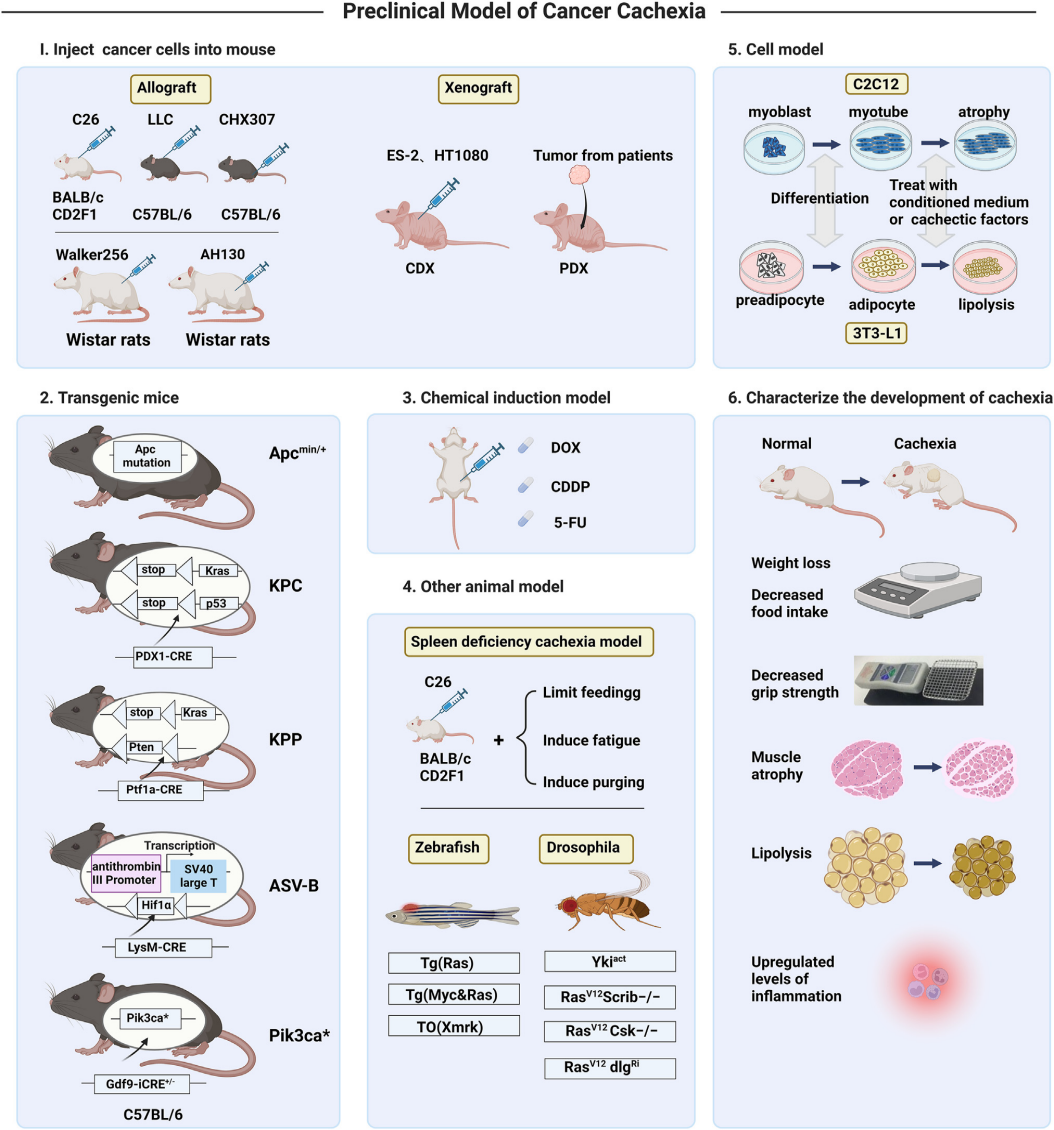
Summary graph of the preclinical model of cancer cachexia.This diagram summarizes the preclinical models currently in common use. Popular cachexia models include inoculated transplant animal models, transgenic mouse models, chemically induced muscle wasting models, other animal models (including zebrafish and Drosophila models), and cellular models. Finally, a summary of the phenotypic characteristics of the cancer cachexia models is presented (Specific details are described in the text). Created with BioRender.com.

Given the heterogeneity and complexity of cancer cells and cancer cachexia and their serious impact on the quality of patient survival, more in-depth studies related to cachexia are urgently needed.^46^ The selection and establishment of a suitable cachexia model is an important guarantee for conducting research, and the current models of cachexia mainly include inoculation and transplantation models, genetically engineered mouse models, and chemotherapeutic drug induction. These animal models have played a key role in revealing the pathogenesis of cancer cachexia and developing therapeutic drugs. However, metastasis is common in patients with cachexia in clinical practice, but there is no animal model that can effectively reproduce the complex metabolic and metastatic processes exhibited by tumors during growth in vivo,^123^, ^124^, ^125^ and the limitations of animal models should be taken into account in future studies, with particular attention given to the fact that animal models cannot fully replicate the complex disease state of humans and that different models have their advantages and disadvantages, so it is important to understand the characteristics of each model in detail and to make a rational choice according to the study objectives to address specific questions.^46^ In summary, in cachexia research, the establishment and selection of appropriate research models are crucial for the prevention and treatment of disease and the development of drugs, and there is still a long way to go in this area, which requires more attention from researchers (Figure 1).

## Author contributions

LL wrote the manuscript; JW and ZH helped in the critical revision; HW and YW conceived the study and edited the manuscript.

## Conflict of interests

The authors declare that they have no conflicts of interest.
